# First Isolation of a Giant Virus from Wild *Hirudo medicinalis* Leech: *Mimiviridae* isolation in *Hirudo medicinalis*

**DOI:** 10.3390/v5122920

**Published:** 2013-11-27

**Authors:** Mondher Boughalmi, Isabelle Pagnier, Sarah Aherfi, Philippe Colson, Didier Raoult, Bernard La Scola

**Affiliations:** 1 Aix-Marseille Univ., Unité de Recherche sur les Maladies Infectieuses et Tropicales Emergentes (URMITE), UM63 CNRS 7278 IRD 198 INSERM U1095, Facultés de Médecine et de Pharmacie, Marseille, France; E-Mails: bougalmi.mondher@gmail.com (M.B.); isabelle.pagnier@univ-amu.fr (I.P.); Sarah.AHERFI@ap-hm.fr (S.A.); philippe.colson@univ-amu.fr (P.C.); didier.raoult@gmail.com (D.R.); 2 Institut Hospitalo-Universitaire (IHU) Méditerranée Infection, Pôle des Maladies Infectieuses et Tropicales Clinique et Biologique, Fédération de Bactériologie-Hygiène-Virologie, Centre Hospitalo-Universitaire Timone, Assistance Publique – Hôpitaux de Marseille, Marseille, France

**Keywords:** Giant virus, *Marseilleviridae*, *Mimiviridae*, *Megaviridae*, *Hirudo medicinalis*, Hirudovirus

## Abstract

Giant viruses and amoebae are common in freshwater, where they can coexist with other living multicellular organisms. We screened leeches from the species *Hirudo medicinalis* for giant viruses. We analyzed five *H. medicinalis* obtained from Tunisia (3) and France (2). The leeches were decontaminated and then dissected to remove internal parts for co-culture with *Acanthamoeba polyphaga*. The genomes of isolated viruses were sequenced on a 454 Roche instrument, and a comparative genomics analysis was performed. One Mimivirus was isolated and the strain was named Hirudovirus*.* The genome assembly generated two scaffolds, which were 1,155,382 and 25,660 base pairs in length. Functional annotations were identified for 47% of the genes, which corresponds to 466 proteins. The presence of Mimividae in the same ecological niche as wild *Hirudo* may explain the presence of the mimivirus in the digestive tract of the leech, and several studies have already shown that viruses can persist in the digestive tracts of leeches fed contaminated blood. As leeches can be used medically and Mimiviruses have the potential to be an infectious agent in humans, patients treated with leeches should be surveyed to investigate a possible connection.

## 1. Introduction

*Hirudo medicinalis* is a leech used medically to extract blood, and it is also valued for its capacity to avoid coagulation by secreting hirudin, an anticoagulant substance discovered in leech saliva by Haycraft in 1884. It took another century for the substance to be successfully isolated and characterized by Markwardt, in 1986 [1]. 

The natural habitat of leeches is mostly fresh water from puddles, and the progressive disappearance of this habitat, as well as the intensive collection of wild leeches for medical purpose in the eighteenth century in Central Europe, led to a dramatic decrease in the population and the concomitant classification of the species on the International Union for Conservation of Nature (IUCN) Red List of endangered species [2]. Collecting wild leeches from natural environments for medical use can be problematic because of they represent a risk for micro-organism transmission. Indeed, in the natural habitat, leeches are in constant interaction with environmental bacteria, protozoa and viruses, which can colonize their digestive tract. The symbiosis of the leeches from the genus *Hirudo* with *Aeromonas* sp. has been well known for years [3], and several cases of human transmission of *A. hydrophila* have been reported when used in microsurgery. In the natural environment, the interactions between leeches and micro-organisms have not been well characterized, even when other associated bacteria have been identified in the animal. Indeed, some *Ochrobactrum* sp., *Bdellovibrio* sp. and *Sphingobacterium* sp. were detected in the bladders of medical leeches [7], and a *Rikenella*-like bacterium was isolated from the gut of a *H. verbena* species [8]. 

The role of leeches as a potential reservoir for viruses has also been suspected. Even if they are not known to be natural carriers of viruses, the persistence of the classical swine fever virus and the Myxoma virus in their digestive tract was demonstrated after leeches were fed with swine and rabbit blood contaminated with those viruses [9,10,11]. In recent years, several other viruses have been demonstrated or suspected to be harbored by leeches [10,11]. In the natural environment, leeches may come into contact with other species of viruses, including the giant viruses that infect amoebae and are now well known to be widely present in aquatic environments and soil [12]. We describe here the first isolation of a giant virus of the family *Mimiviridae* from a wild specimen of a medical leech, *Hirudo medicinalis.*

## 2. Results

### 2.1. Virus isolation and preliminary characterization

**Figure 1:**
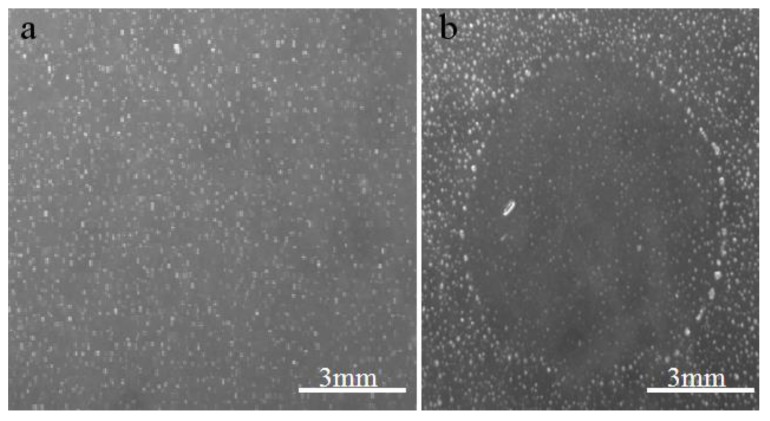
Aspect of agar plate on which samples were inoculated. (a) negative control, (b) Hirudovirus starin growth leading to lysis of amoebas.

**Figure 2:**
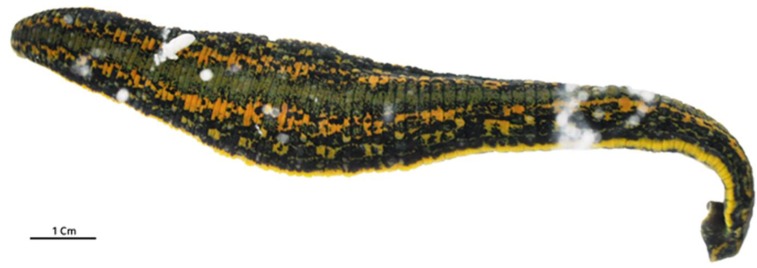
*Hirudo medicinalis* in which Hirudovirus strain was isolated.

**Figure 3:**
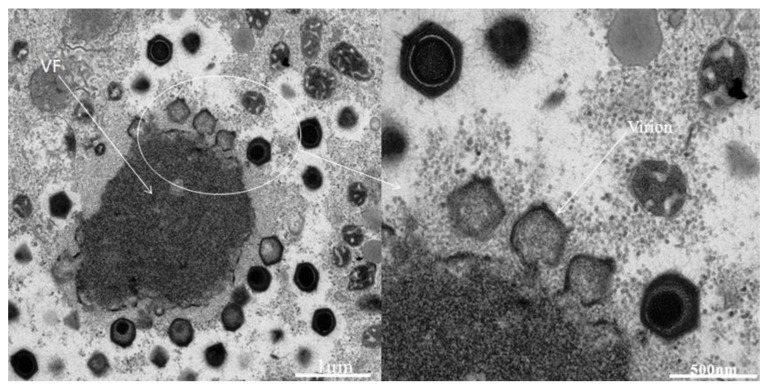
Typical aspect if Hirudovirus virus factory (VF) growing in *A. polyphaga.*

The inoculations on amoebae in agar plate showing lysis plaque (Figure 1) resulted in the isolation of one single virus from one Tunisian *Hirudo medicinalis* (Figure 2) collected at “Oued Sarrath” (35°46'0" N, 8°37'0" E). No virus was isolated from PAS buffer used to rinse leeches in order to remove ethanol. The virus was isolated from the internal organs and digestive tract Electron microscopic examination of the negative-stained culture supernatant showed the typical icosahedral structure of giant viruses, with a capsid (410 nm±10) surrounded by long fibrils (110 nm±10) and an overall viral particle diameter of 520 nm±10. Transmission electronic microscopy showed a typical aspect of virus factory within *A. polyphaga* 12h post infection (Figure 3). Based on this observation, we pre-classified the virus in the family *Mimiviridae*. This new strain of Mimivirus was named Hirudovirus.

### 2.2. Genome sequencing, annotation and comparative genomics

**Figure 4:**
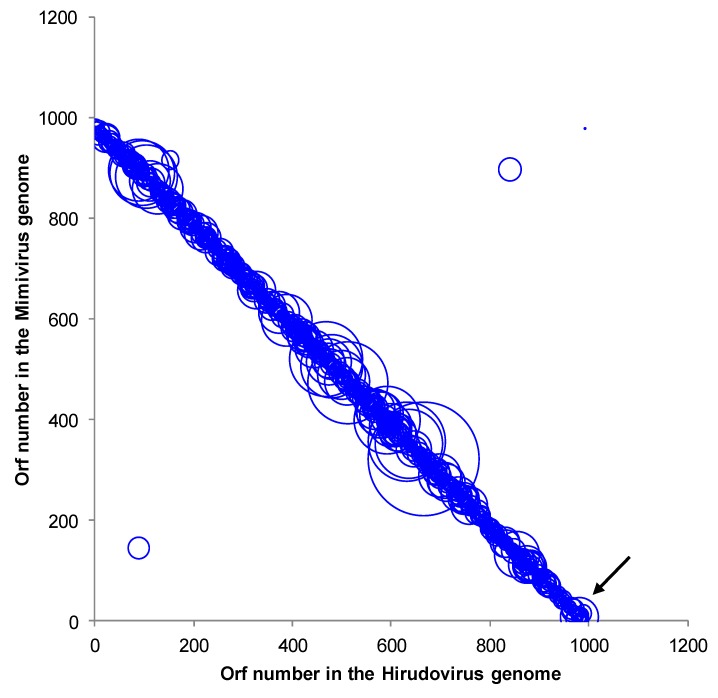
Genomic dotplot between Mimivirus and Hirudovirus pairs of orthologs by BLASTp analysis. The diameter of the circles is proportional with the BLASTp bit score. The arrow indicates the position between the two contigs.

The sequenced genome assembly resulted in two scaffolds of 1,155,382 and 25,660 base pairs, with a low (28%) mean GC content, which is similar to those of other mimiviruses of amoebae [13]. A total of 992 predicted genes, ranging in size from 34 to 2,959 amino acids, were detected in the draft genome of the strain Hirudovirus. This corresponds to a protein coding density of 0.9 genes/kilobase pair similar to values determined for Mimivirus (0.83) and Mamavirus (0.86), which belong to lineage A of amoebal mimiviruses [13,14,15]. A functional annotation was identified for 47% of these genes (corresponding to 466 proteins). The comparative genomic analyses showed that the Hirudovirus strain genome shared the highest number of *bona fide* orthologs with Mimivirus (n= 806; 82% of the Hirudovirus gene content) and Mamavirus (805), whereas 435, 402, 361 and 338 *bona fide* orthologs were found from other mimiviruses of amoeba including *Megavirus chiliensis* [16], LBA111 [17], Moumouvirus [18] and Monve [12], respectively. Besides, 38% of the Hirudovirus predicted proteins had significant BLASTp hits from organisms outside the family *Mimiviridae*. Of the Hirudovirus predicted proteins, 774 (78%) and 190 (19%) found a protein from Mimivirus or Mamavirus, respectively, as top hit in the GenBank non-redundant database. Proteins from other mimiviruses were identified as top hits for other Hirudovirus proteins, and among them, 18 were detected in the genome of Lentillevirus, which is classified within lineage C of amoebal mimiviruses [19]. Four Hirudovirus predicted proteins were not found by BLASTp in Mimivirus and Mamavirus but were found in other mimiviruses of amoeba; especially, all four were found in Courdo7 virus, a mimivirus of amoeba from lineage C [13]. Finally, two Hirudovirus proteins (HIRU_S223 and HIRU_S244) had no significant BLASTp hit in the NCBI Genbank non-redundant and environmental protein sequence databases but BLAST search using nucleotide sequences detected hits in the Mimivirus and Mamavirus genomes. The Hirudovirus and Mimivirus genomes are highly similar and collinear (Figure 4). *Bona fide* orthologs from these viruses showed a mean amino acid identity of 99.1±2.6% (range, 72.1-100%), respectively. In addition, mean nucleotide identities for these *bona fide* orthologs was 99.3±1.8% (range, 80.7-100%), respectively. These values are in the same order of magnitude than those described for b*ona fide* orthologs of Mimivirus and Mamavirus that is another strain of Mimivirus [15]. 

### 2.3. Phylogeny

**Figure 5:**
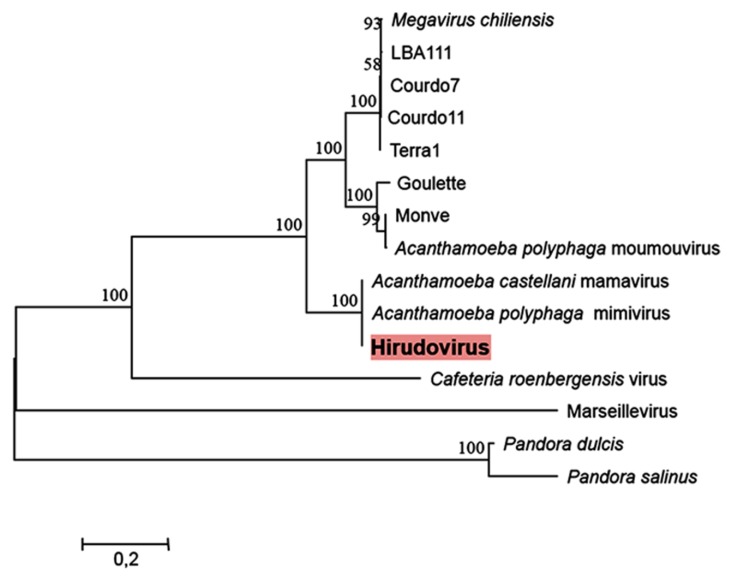
Maximum likelihood phylogeny reconstruction based on a concatenated alignment of the VVA18 helicase, the DNA polymerase B family and the VV-A32 packaging ATPase. Phylogeny reconstruction was performed using the MEGA5 software on a total of 1835 positions in the final dataset. Bootstrap values (as a result of 1,000 replicates) are shown as percentages next to the branches. A discrete Gamma distribution was used to model evolutionary rate differences among sites. The tree is drawn to scale, with branch lengths measured in the number of substitutions per site.

Phylogenic reconstructions based on a concatenated alignment of three conserved genes shows that Hirudovirus strain clusters with Mimivirus and Mamavirus in the lineage A, previously defined for mimiviruses of amoebae [13], which agrees with the results of the comparative genomic analyses (Figure 5).

## 3. Discussion

One single specimen, among the 5 *Hirudo medicinalis* analyzed, led to the isolation of a giant virus from the internal part of the animal. The virus was absent from the flesh, and the presence of the virus in the internal organs and the gastrointestinal tract suggests that the giant virus may have been ingested with food. Indeed, giant viruses can naturally be found in several ecological niches, such as fresh water, seawater (including hypersaline environments), and soil [12,20,21]. Hence, the presence of giant viruses in the same ecological niches as wild *Hirudo* species can explain the presence of a mimivirus in the digestive tract of the leech. Several studies have shown that viruses persist in the digestive tract of leeches fed with contaminated blood [3,5,6,9]. Moreover, leeches are known to exhibit symbiosis with several bacteria, mainly those species from the genus *Aeromonas*, and with several proteobacterial genera [8,9,20]. This symbiosis becomes problematic when leeches are used in a medical context and infected animals eject symbiotic bacteria from their digestive tracts into the bite. Indeed, feeding leeches blood from animals treated with antibiotics can lead to the acquisition of resistance in the symbiotic bacteria, and this complicates the treatment of patients in case of accidental infection through hirudotherapy [7]. Alternatively, we cannot exclude the possibility that mimiviruses can be contaminant of water in hospital aquaria used to grow leeches. Further studies should examine this potential niche because giant viruses have already been isolated from aquatic environments (including some that are close to humans, such as hospital water). Another concern stemming from the presence of amoebal mimiviruses in leeches is that a growing body of evidence suggest that these viruses could be a rare cause of pneumonia [17]. Notably, mimiviruses of lineage C were recently isolated from the bronchoalveolar fluid and the stools of Tunisian patients with pneumonia. Additionally, a mimivirus of lineage A, a close relative to the strain Hirudovirus, was isolated from the contact lens fluid of a young woman exhibiting keratitis [19]. Our work does not find evidence of pathogenic effects of the Mimivirus on the leech. However, further investigations should be performed to evaluate the impact of infection on the viability of these medically important animals. The analysis of the 1.18 megabase genome of the strain shows gene content closely related to Mimivirus and Mamavirus, which belong to lineage A of amoebal mimiviruses [13]. Notably, 97% of the Hirudovirus genes were most similar (via BLASTp) to proteins from either Mimivirus or Mamavirus, which is another Mimivirus strain and Hirudovirus and Mimivirus genomes showed high levels of similarity and collinearity. Moreover, these findings are consistent with those of the phylogenetic analysis. Hirudovirus strain is the first giant virus to be isolated from a member of the family *Hirudinae*. However, other mimiviruses have been isolated from humans, whereas marseilleviruses, other giant viruses that infect amoebae, have been isolated from humans and *Eristalis tenax,* an insect member of the order *Diptera* [22]. These data suggest that the diversity of the ecological niches for these giant viruses is not yet fully understood. 

## 4. Materials and methods

**Samples and inoculation of amoeba.** Animals tested for the presence of giant viruses came from two small streams at two locations in Tunisia and France. A total of five leeches of the species *Hirudo medicinalis* were isolated*,* three in Tunisia and two in France. Wet bottles were used for transporting live samples to the laboratory. The method used to prepare the animals and the procedure for isolating the virus have been recently detailed elsewhere [20]. Briefly, after morphological identification, *Hirudo medicinalis* were rinsed with 96% ethanol for 20 min and then washed with sterile PAS (Page’s amoebal saline). The washing solution was saved. The animals were dissected to obtain samples of flesh (F) and internal organs in digestive tract (IO+DT). These samples were crushed mechanically before inoculation using the high throughput isolation system previously described [21].

**Preliminary characterization of viruses.** After the appearance of an amoebal lysis plaque, indicates the presence of a giant virus, the agar plate was cut into small pieces around the plaque, resuspended in 500 µl sterile PAS, centrifuged at 15000 rpm for 30 minutes, and re-inoculated onto a fresh amoebal monolayer. The presence of the giant virus was then assessed by Hemacolor staining after cytocentrifugation of a 100 µl sample of culture supernatant. The preliminary classification of the virus was performed by observation of the culture supernatant, which was negative stained with a 3% ammonium molybdate solution and evaluated with electron microscopy. 

**Whole genome sequencing.** For whole genome sequencing, 25 culture flasks (150 cm^2^) were prepared with 5 ml of virus, 10 ml of amoebae and 30 ml of nutritive medium PYG (Proteose peptone, Yeast extract, Glucose). After amoebal lysis, viruses were purified and concentrated; genomic DNA extraction was performed with phenol-chloroform, and the whole genome was pyrosequenced on the 454-Roche GSFLX as previously described [23].

**Sequence assembly, gene prediction and annotation.** The genome assembly was performed with the Newbler software [24]. Genes were predicted using the GeneMarkS program [25]. All ORFs smaller than 100 codons were considered only if they had a significant hit (BLASTp e-value <1e-4, both query and target coverages >25%) in the NCBI GenBank non-redundant protein sequence database (nr), including genes from *Acanthamoeba polyphaga* Mimivirus [26], *Acanthamoeba castellanii* mamavirus [15], *Acanthamoeba polyphaga* moumouvirus [18], LBA111 [17], *Megavirus chiliensis* [16], Moumouvirus monve and the recently described *Pandoravirus dulcis* and *Pandoravirus salinus* [27]. The Hirudovirus predicted proteins were additionally searched by BLASTp against the NCBI environmental protein sequence (env_nr) and non-redundant nucleotide sequence (nt) databases. Next, genes were annotated using comparative genomics, including BLASTp searches against the GenBank nr database and the published gene repertoires of mimiviruses, and *P. dulcis* and *P. salinus*. *Bona fide* orthologs, which are orthologs identified through the widely used approach consisting in the identification of reciprocal best hits between two gene repertoires, were determined here by the Proteinortho program with an e-value cut-off of 1e-3 and minimum amino acid identity and coverage of 30% and 70%, respectively [28]. In addition, a search was performed for all of the orthologous genes shared with the previously cited viruses using BLASTp with an e-value threshold of 1e-4 and minimal coverages of query and target sequences of 25%. Predictions of transfer RNA (tRNA) were performed using the ARAGORN software [29]. 

**Phylogenetic analysis.** The phylogenetic analysis was based on three concatenated core genes (the VV-A18 helicase, the VV-A32 packaging ATPase and the B family DNA polymerase) of the nucleocytoplasmic large DNA viruses [14]. Multiple sequences alignments were performed using the MUSCLE program [30], informational positions were selected by the Gblocks tool [31] and a phylogenetic tree was constructed using MEGA5 [32] and the maximum likelihood method. Genes from Marseillevirus and pandoraviruses were used as outgroups.
